# Health equity, care access and quality in headache – part 1

**DOI:** 10.1186/s10194-024-01712-7

**Published:** 2024-01-29

**Authors:** Claudio Tana, Bianca Raffaelli, Marcio Nattan Portes Souza, Elena Ruiz de la Torre, Daniel Gams Massi, Najib Kisani, David García-Azorín, Marta Waliszewska-Prosół

**Affiliations:** 1 Center of Excellence on Headache and Geriatrics Clinic, SS Annunziata Hospital of Chieti, Chieti, Italy; 2 Department of Neurology, Charité – Universitätsmedizin Berlin, corporate member of Freie Universität Berlin and Humboldt Universität zu Berlin, Berlin, Germany; 3 Clinician Scientist Program, Berlin Institute of Health (BIH), Berlin, Germany; 4 Department of Neurology, Hospital das Clínicas da Universidade de São Paulo, São Paulo, Brazil; 5 European Migraine and Headache Alliance, Brussels, Belgium; 6 Neurology Unit, Douala General Hospital, Faculty of Health Sciences, University of Buea, Buea, Cameroon; 7 Department of Neurology, Mohammed VI University Hospital, Marrakech, Morocco; 8 Headache Unit, Department of Neurology, Hospital Clínico Universitario de Valladolid, 47003 Valladolid, Spain; 9 Department of Neurology, Wroclaw Medical University, Wroclaw, Poland

**Keywords:** Migraine, Health equity, Care access, Stigma, Clinical governance, COVID-19

## Abstract

Current definitions of migraine that are based mainly on clinical characteristics do not account for other patient’s features such as those related to an impaired quality of life, due to loss of social life and productivity, and the differences related to the geographical distribution of the disease and cultural misconceptions which tend to underestimate migraine as a psychosocial rather than neurobiological disorder.

Global differences definition, care access, and health equity for headache disorders, especially migraine are reported in this paper from a collaborative group of the editorial board members of the Journal of Headache and Pain. Other components that affect patients with migraine, in addition to the impact promoted by the migraine symptoms such as stigma and social determinants, are also reported.

## Introduction

Migraine is a complex neurological disorder that involves not only neurobiological symptoms but also multiple domains for each patient (e.g. psychosocial, personal and economic). Current definitions of migraine are based on the occurrence of attacks of moderate to severe, throbbing and pulsating pain on one side of the head, exacerbated by physical exercise and associated with nausea/vomiting and/or photo/phonophobia. This definition does not account for other patient’s features which are no less important, such as those related to an impaired quality of life due to loss of social life and productivity [1]. Also, the classic definition does not evaluate the differences related to the geographical distribution of the disease, which is not attributable only to the geographic nuances inherent in each country, but also to the differences in terms of estimation method, the definition that is used for diagnosis but also cultural misconceptions which tend to underestimate migraine as a psychosocial rather than neurobiological disorder [2].

The Global Burden of Disease (GBD) study estimates the global prevalence of migraine to be around 14% (95% CI 12.9–15.2) and of active headache disorders around 52% (48.9–55.4). The GBD 2021 study is not based on a direct data surveillance system and relies primarily on secondary data collected by a broad network of government officials, medical professionals and scientists [3]. However, the vast majority of studies have been conducted in high-income countries, therefore estimation bias can account for the difference of epidemiology across the different population groups [3].

The recent pandemic from the novel coronavirus disease 2019 (COVID-19), the introduction of effective vaccines and the recent conflict-related economic crises have contributed to change the definition of migraine from a single headache disorder to a complex multifaceted disease, and to the different geographic framework [4].

This document, written by a collaborative group of the editorial board of the Journal of Headache and Pain coming from multiple geographical areas, aims to report the differences in terms of definition, care access, and health equity for headache, especially migraine and secondary headaches. We also addressed challenges in headache medicine based on the experience of the COVID-19 pandemic. Stigma and social determinants of migraine are also described. Part 1 is an introduction to continue our discussion in Part 2, which will cover the organization of headache services with a focus on challenges in low-, middle- and high-income countries. Our other goal is the desire to draw attention to pressing issues in headache medicine and to spark academic, social, and political discussion to improve patients’ lives.

## Epidemiology

### Geographical/geoeconomical differences

About half of the global population is afflicted by an active headache disorder, predominantly tension-type headache (TTH) and migraine [3, 5]. Prevalence statistics, drawn from a plethora of epidemiologic studies, are nonetheless limited by methodologic issues [4]. These include differences in case definitions used, sampling method, and geographic particularities inherent in each country. An interesting observation is that migraine prevalence is reported to be highest in Nepal – a phenomenon that might be linked to the country’s altitude – and lowest in China [6, 7]. For TTH, Afghanistan and Brazil have the highest prevalence, while China has the lowest rate [6].

The geographic differences are further highlighted in the GBD study, which found a lower headache prevalence in Southeast Asia, East Asia, Oceania (SEEAO), and Sub-Saharan Africa (SSA), compared with high-income countries [6]. The latter accounts for about 15% of the global population, but most of the available epidemiological data is derived from these countries and few prevalence studies are available from low- and middle-income countries (LMIC) [3]. The underrepresentation of data from LMIC invites a measured interpretation of the prevalence estimates. This observation also highlights an urgent need for epidemiological research in LMICs.

In tandem with geographic variations in prevalence estimates, one must also account for the profound influence of geo-economic and cultural factors [5]. These forces shape societal attitudes toward headache disorders and, in part, dictate the availability, accessibility, and affordability of headache care. People in LMICs face considerable challenges related to the recognition and management of headache disorders, a struggle amplified by limited economic resources, overburdened healthcare services, and a dearth of specialized clinics [5]. Cultural misconceptions can further muddy the waters. For instance, migraine is often regarded as a psychosocial rather than neurobiological disorder in emerging economies such as China and India [8, 9]. This, in turn, hampers the delivery of appropriate treatment and allocation of adequate headache care services.

The economic ramifications of headache disorders have primarily been studied in high-income countries, with direct costs for migraine management exceeding $1000 per person per year [10, 11]. It is also worth mentioning that indirect costs due to productivity losses are more than double [10, 12, 13]. Unfortunately, comparable data for LMICs is conspicuously sparse. Available estimates from nations like Russia, Zambia, and China hint at a similar economic burden, with indirect costs amounting to about 2% of their Gross Domestic Product [14–16].

Cost-efficiency can be improved through strategic reorientation of healthcare resources towards headache management, complemented by education interventions, especially in low- and middle-countries [5], where the self-management with simple analgesics has been by far the most cost-effective strategy for migraine treatment and represents a highly efficient use of health resources. Predictive modeling for China, India, Russia, and Zambia suggests that acute treatments using simple analgesics might offer the greatest cost-benefits among all therapeutic interventions [17].

To confront the geographic and geo-economic disparities in headache care, a thorough overhaul of existing healthcare policies and an increased allocation of resources for headache disorders in LMICs is much needed. This recalibration, aimed at improving patient outcomes and reducing the economic burden, should incorporate the development of sustainable healthcare models that prioritize comprehensive management of headache disorders.

## Environmental and occupational issues based on the example of the COVID-19 pandemic

### Headache and pandemics/syndemics

The recent widespread diffusion of a novel coronavirus (SARS-CoV-2) has radically changed the epidemiological, clinical and diagnostic scenario of several disorders, especially headache [18]. In the context of the pandemic from the novel coronavirus disease 2019 (COVID-19), headache can be a clinical manifestation of the acute form of the disease, or the persistent, difficult-to-treat and disabling symptom of post-acute sequelae of SARS-CoV-2 infection (also known as long COVID headache) or can act as comorbidity with COVID-19, with a synergy effect on clinical picture in term of intensity of pain and refractoriness to the treatment [19, 20]. The different geographical spread of the pandemic, at least in the first wave, has modified the different geographical distribution of symptoms such as headache, resulting in a higher prevalence of headache also in countries where the prevalence was lower before the pandemic (e.g. China) [21]. The aggregation of two or more disease clusters is defined as syndemic and the synergistic epidemic effect has a significant impact on biological and prognostic consequences and the whole disease burden, for almost all disorders and headache and related pain syndromes are not spared from this phenomenon [22].

Several data report how patients with migraine and co-infection from SARS-CoV-2 complain of more intense pain which is often less responsive to common analgesics [23]. Patients with a migraine history and a migraine-like phenotype can respond well to triptans, while those with a TTH-like phenotype can be improved by single doses of paracetamol [24]. A good response rate in term of pain relief has been obtained with indomethacin in patients with the migraine-like phenotype, however several side effects are associated with its long-term use (e.g. cardio and nephrotoxicity) limits significantly the prescription in the daily routine [25]. In long COVID, the use of prophylactic drugs such as amitriptyline has been associated with a significant reduction of migraine crises in 44% of cases, therefore its use has been recommended in those patients experiencing 4 or more headache days per month [26]. Few evidence is showing a good response rate to the sphenopalatine ganglion block in terms of headache days reduction however the very limited data does not recommend its use in the daily routine [27].

The recent introduction of migraine prevention drugs such as those targeting the Calcitonin Gene Related Peptide (CGRP), has given new options for the treatment of migraine. However, due to the high cost and low availability in all countries, their use is limited to the cases that are refractory to the other preventive treatments for at least 8 weeks of therapy and actually they are available only in high-income countries [28, 29]. CGRP mAbs treatment is not associated with the risk of severe COVID-19 outcomes or positive results at the SARS-CoV-2 test therefore its use is safe for migraine prevention also during the pandemic [30]. However, no studies have so far investigated to effectiveness of these drugs in long-term COVID headache, therefore actual evidence derives from the migraine prevention trials during the SARS-CoV-2 pandemic [30].

While it has been established that patients with a history of migraine do not have a significant risk increase of developing COVID-19, the biological and immunological mechanisms underlying the effective interaction between the two disorders and their differences according to genetic variability across different countries are largely unknown [31]. The SARS-CoV-2 binding to the angiotensin-converting enzyme 2 (ACE2) binding from SARS-CoV-2 is associated with a reduced anti-inflammatory effect due to enzyme activity reduction, in favor of inflammatory headache [32]. Less evidence argues in favor of hypoxia, hypercapnia and of the direct neuronal tissue invasion from SARS-CoV-2 [33]. Persistent immune system activation from cytokine hyperproduction, unresolved neuronal damage or meningeal inflammation may account for the persistent headache in long COVID, and other neurological manifestations such as cognitive deficits and brain fog may be related to an glutamate hyperproduction and upregulation of N-methyl-D-aspartate (NMDA) receptors [24].

Gut dysbiosis in combination with genetic predisposition seems to modulate migraine attacks, and some theories suggest that the microbiome-gut-brain axis can link not only to neurodegenerative and neurodevelopmental diseases [34–36] but also to other potentially reversible neurological diseases, such as migraine and long COVID headache [36–38].

Promotional measures of lifestyle changes, a balanced diet and regular physical activity can modulate effectively the gut microbiota composition, but are extremely difficult to reach in low-income countries, making even more clear the geographical difference [39, 40].

### COVID-19 and vaccines

Another important aspect related to the COVID-19 pandemic which has changed the phenotype of migraine in recent years has been the introduction of vaccines. At the start of pandemic, the problem has been even more complex due to the different accessibility to vaccines from the various countries. The first vaccines were introduced in mid-2020 and several formulations based on different mechanisms of action were developed [41]. Traditional vaccines contained inactivated viruses or recombinant viral vectors and more advanced vaccines were based on mRNA or viral vectors. The vast majority of adverse effects (AE) after vaccination are mild and resolve within a few days. Severe adverse reactions such as anaphylaxis, thrombosis, carditis, and severe polyneuropathy have been observed rarely [42].

A meta-analysis based on 1.57 million people showed that headaches were the third most common AE and occurred up to 7 days after vaccination. They were observed in 22% and 29% of individuals after the 1st and 2nd doses of vaccination, respectively. There was no difference in the incidence of headaches depending on the type of vaccine and the percentage of headaches after 1 dose decreased with age [43]. The phenotype of vaccine-related headache resembled migraine-like in about 30%, and the headache was throbbing with sensory hypersensitivity and intensified during physical activity. It was shown that the incidence of headache after vaccination was higher in individuals with a history of headache [44]. In a group of 841 migraine patients, it was shown that more than 60% of both the first and second doses of the vaccine were followed by a migraine attack that lasted longer, was more severe and was more refractory to treatment [45]. It has also been described as a case series of patients with cluster headache who, after a long period of remission, developed a new cluster with frequent attacks early after vaccination. In addition, the new cluster episodes occurred in them at different times of the year than usual [46] Some reports of cluster headache occurring de novo immediately after vaccination have been described [47–49].

In most cases, acute headache following vaccination is associated with the immune response triggered by the vaccine, and in this regard, there are more similarities than differences with post-COVID headache [50]. However, post-vaccination headaches can also be associated with severe AEs and, in certain situations, should be a red flag that may suggest secondary headaches in the course of, for example, cerebral venous thrombosis (CVT) or other thrombotic complications in the background of immune thrombocytopenia [51]. Such situations can be particularly insidious in patients with chronic headaches in whom prolonged headache after vaccination may be trivialized [50]. The widespread vaccination against SARS-CoV-2 has radically improved the outcome of patients with COVID-19 and has been associated with a significant overall reduction in mortality. The majority of side effects are mild and transient, therefore periodic vaccination against the SARS-CoV-2 virus is strongly recommended, especially in some at-risk categories of unfavorable outcomes such as immunocompromised patients and older adults [52].

### Long COVID

As mentioned above, another important consequence of the pandemic is the persistence of post-COVID-19 syndrome for which the term long COVID syndrome is commonly used [53]. Despite unclear diagnostic criteria, it implies the persistence of significant symptoms from 4 to 12 weeks after an infection. Neurological symptoms are frequently observed and the most common include headache, cognitive impairment, brain fog, fatigue syndrome and neuropsychiatric disorders like depression and sleep disturbances [54–56]. Other symptoms include cardiovascular, pulmonary/respiratory or musculoskeletal disorders [55]. The symptoms and course of long COVID vary depending on the viral variant that caused the infection [57, 58].

Long COVID headache may manifest as either a worsening of the course of a pre-existing headache or the appearance of a new headache, which is most often accompanied by other symptoms of infection such as hyposmia or anosmia [19]. Most frequently, it has a tension-type phenotype, less often a migraine-type. It manifests with bilateral, compressive pain without additional symptoms. The new headache may be daily and persistent and its phenotype may mimic new daily persistent headache (NDPH) [59]. The presence of headache during the acute phase of infection has been associated with a better prognosis and low mortality from COVID-19. This difference most likely indicates the activation of the immune response during acute viral infection [32, 60].

Patients with a history of previous headache usually report an increase in the frequency and intensity of headaches. Furthermore, patients with refractory headache in the acute phase of infection seem to have a higher incidence of long COVID headache [61].

Long COVID symptoms are still an object of observation and scientific research because most of them require observation over time. In addition to the long COVID headache described in this chapter, post COVID symptoms can involve many systems and organs. We do not have precise data on geographical differences between long-covid symptoms. Single studies have shown no differences in the distribution of these symptoms between residents of metropolitan-urban residences and regional-urban/rural residences. To date, a worse prognosis of long COVID symptoms, especially fatigue, has been shown to correlate with age, female gender and hospitalization associated with a more severe course of the disease [21, 22, 32, 58–61].

## Stigma and social determinants

### Stigma

Patients with migraine quite often have to lead with more than the impact promoted by the several symptoms of the recurrent migraine attack. Migraine stigma is a common problem that adds to the suffering of this condition. Stigma can be defined as the mark or condition or status that undergoes to social devaluation, and it is described from three different perspectives: public, structural, and internalized stigma [62].

Public stigma refers to the general idea of stereotypes that circulate in a determined society in a certain period. It is revealed by several labels commonly used to describe migraine, such as *malingerer*, *pill popper*, *hysterical, drug-seeking*, *lazy* or *person incapable of handling stress* [63]. The idea of an easily solved disorder, that happens predominantly to wealthy women “who can afford to lie in the bed” is nourished by several cultural manifestations, from literature to pharmaceutical advertisements, and media representations [64]. There is a public conviction that people with migraine “have very little resistance, or are using it as an excuse” [65]. One of the social consequences of this misrepresentation of migraine is that the population that does not correspond to this stereotype frequently will fail to recognize the migraine symptoms in themselves, and this might delay seeking for healthcare attention [64]. Public stigma is often rooted in a lack of awareness about the condition. Public stigma is not always overt, and it can be subtle. For example, people with migraine may feel uncomfortable talking about their condition at work, because they are worried about being judged.

Structural stigma refers to the consequences imposed by migraine stigma in the social structure, such as laws and health policies. It may manifest in different structures of society. One example is the relatively limited mean time dedicated to headache training observed worldwide in medical education, which is limited to no more than 4 hours in undergraduate education, and no more than 10 hours for medical specialists [66]. Another example is the discrepancy between the prevalence and impact of migraine and the rate of funding dedicated to migraine research. In 2009, if considered the burden related to headache diseases and compared to other conditions, the funding expected by National Institute of Health (NIH) would be more than $103 million/year, whereas the amount received was between $6.8 and $13 million/year [67]. One of the consequences of this discrepancy is the lack of opportunities to develop research, and a lowering status of headache medicine in medical departments, when compared to other areas of the neurological field [64]. Structural stigma is also revealed by the low rates of search for help by migraine patients. One study evaluating patients with chronic migraine, presenting with migraine attacks for more than 15 days per month for at least 3 months, showed that no more than 40.8% of patients have ever sought healthcare attention due to the headache [68]. The workplace is a common field of structural migraine stigma. In a survey from 2016 with 4024 adults, half of those who called out of work for a headache reported they did not reveal to their supervisors the reason for absenteeism. Furthermore, half of the managers surveyed did not consider headache as an acceptable reason to leave work, showing how clear is migraine stigma in the workplace [64].

Internalized stigma in migraine is the negative perception of oneself that is held by a person with migraine. It can lead to feelings of shame, guilt, and isolation. It is a consequence of the absorption by patients with migraine of the assumptions created about their condition. Internalized stigma may impose serious consequences on patients’ self-esteem and mental health [69]. In research by Young et al. that compared the stigma between patients with epilepsy, chronic migraine, and episodic migraine, those with chronic migraine presented the most severe scores, and impaired ability to work was the strongest predictor of stigma [70]. Migraine stigma might also affect patients’ family relationships. Over a third of patients with chronic migraine reported it affected their relationship with their partner, and 71% reported that they would be better parents if they did not have headaches [71]. One of the worst consequences of the internalized stigma is the impairment of the ability to recognize migraine as a disease and seek help.

Migraine stigma is also affected by the gender gap, imposing on women a disproportionately higher burden than men, and not only because of the higher prevalence. Often women are taken less seriously by healthcare providers, and headache might be “psychologized”. Women have less access to adequate treatment and are more likely to report medication overuse headache (MOH) [72].

In conclusion, the stigma of migraine is a complex and multifaceted problem that can have a significant impact on the lives of people with this condition. It can lead to discrimination in the workplace, healthcare settings, and personal relationships. It can also lead to feelings of shame, guilt, and isolation. It affects women more than men. Some promising approaches to chasing migraine stigma include raising awareness about migraine and their impact on people’s lives, challenging negative stereotypes, and providing support for people with migraine. Reducing the stigma of migraine is an important step towards improving the quality of life for people with this condition. By doing so we can create a more inclusive society where patients with migraine are treated with respect and dignity.

### Social determinants

In recent years, a growing attention has been made to the social determinants (SD) of health i.e., the conditions in which people are born, live, grow up, and function, and which affect their access to health and outcome [73]. As early as 1848 Rudolph Virchow noted that “*if medicine is to accomplish its great task, it must intervene in political and social life*” [74]. The World Health Organization (WHO) currently divides SD into 5 core domains that affect human health: 1. access to and quality of health care, 2. access to and quality of education, 3. social context, 4. economic stability and 5. environment/neighborhood [75].

Despite significant advances in the diagnosis and treatment of headaches, many patient groups in the world are still marginalized for historical, social or economic reasons. These include communities of color, people experiencing poverty, the un- or under-employed, the un- and under-insured, immigrants, women, people with low levels of education. These groups are also underrepresented in migraine research [76, 77].

According to the analyses from the 2017 GBD, the disability-adjusted life years (DALYs) are significantly higher for headaches in women compared to men. In women, migraine is among the top five most common causes of disability [6, 78]. The course of migraine in women is undoubtedly influenced by the nature of the pain experience, involvement in multiple fulfilling roles, and different coping strategies [79]. On the other hand, men are less likely to seek medical care and access treatment, which is often due to a certain perception that men are the gender that must be strong. In addition, the higher prevalence of migraine in women has contributed to the classification of the condition as a “female disorder” which has negative consequences for both sexes [80].

In addition, migraine prevalence is higher (regardless of gender) among those with lower family income and the unemployed, as well as the elderly and disabled [81].

According to the study results from Loder et al., racial and ethnic minorities in the U.S. may not receive adequate medical care for headache treatment compared with whites [82]. Even after accounting for demographic and insurance differences, black people are 40% less likely to be treated by a neurologist than whites. On the other hand, they are more likely to end up in emergency departments due to undertreated migraine [83]. The role of structural racism has been raised during the COVID-19 pandemic where disproportionate morbidity and mortality has been reported in Latinos, Asian, African-American and black populations. The key factor was the impact not of race alone but of access to health care [84]. The literature is very sparse when it comes to pediatric populations, but it has been shown that white children are significantly more likely to have neuroimaging for headaches than children of other races [85]. However, the prevalence of headache in children from all latitudes remains underestimated [86].

Migraine disproportionately affects also those living in poorer social conditions in terms of prevalence and severity and result in higher stress due to exogenous factors such as personal and food insecurity, employment, poverty and poorer access to health care [87, 88]. U.S. studies show that low-income or uninsured individuals who lack opportunities for proper medical care are more likely to develop medication overuse including opiates [89].

The etiology of migraine is not well understood but it is hypothesized that is a multifactorial disease influenced by numerous factors including genetic, epigenetic but also lifestyle, personal history and environmental factors [76, 90]. People living in areas with high air pollution, exposure to toxins, lack of access to water, frequent changes in weather conditions, and poor nutrition experience greater psychosocial stress, which affects the course of the disease [91, 92].

All of the above social determinants affect specific areas of disparity and/or inequality in adult and pediatric headache. These are primarily the use of health care services for migraine treatment, more frequent misdiagnosis, lack of trust in health care professionals, inadequate treatment and increased risk of migraine progression and burden of the disease [5].

Analyzing social determinants and all the above aspects, it should be remembered that the primary factors for the limitations in resource-restricted settings are as political instability, ongoing wars, limited resources, and mass displacement of people.

### Patients’ point of view

Patients are the individuals directly experiencing migraine and its associated stigma. By considering their perspective, physicians could gain a holistic understanding of the impact of stigma on their lives. This insight goes beyond clinical observations and provides a comprehensive view of the challenges they face in their daily lives, including social, emotional, and psychological aspects [65]. Understanding patients’ perspectives is fundamental to providing patient-centered care. It allows healthcare professionals and researchers to tailor interventions, support systems, and educational materials to better meet the needs of individuals living with migraines. This patient-centered approach can improve the effectiveness of interventions and ultimately enhance the quality of care. Patients’ organizations, such as the European Migraine and Headache Alliance (EMHA), are critical in shaping policies related to healthcare, disability accommodations, and anti-stigma initiatives.

During 2023, the EMHA ran a survey about the stigma of people with migraine in different areas, e.g., relationships with friends, family, workplace or doctor’s appointments (EHMA survey 2023, unpublished data). In total, 4.210 responders from 17 European Countries participated in the survey. The vast majority were women and most responders reported to have severe migraine with more than 8 attacks per month. Overall, 93% of survey participants believe that migraine is not understood by the general public and it is more stigmatized than dementia, Parkinson’s disease or stroke. One of the most frequently reported reasons for stigma is that migraine is often considered as “just a headache” and affected individuals are seen as weak, complaining people [65, 93].

Many of them feel stigmatized at their workplace, and 62% of them feel that migraine has affected how their employer assesses their value. They feel anger, loneliness and sadness at their workplace because of the stigma they experience. As a result, they feel uncomfortable disclosing their condition, they are afraid of being “punished” and they try to hide their disease at work. Even more worryingly, 35% of responders also felt stigmatized by their doctor, often avoiding or delaying seeking treatments due to concerns about what their doctor may think about them.

Similar topics emerged also from a US-American patients’ report in a focus group format [94]. Similar to their European counterparts, key topics from the patients’ point of view were the impact of migraine on family and work, misunderstanding by others, and issues related to medical care, such as feeling dismissed by the treating physicians [94].

Stigma in migraine often comes due to a lack of understanding of the condition and to its invisibility [95]. The debilitating nature of migraine cannot be immediately seen or recognized from the outside and patients often live with the fear of not being believed [95]. Among patients with chronic migraine, almost half had the feeling that even their spouse did not believe them about having headache attacks [71].

From the patient’s point of view, there is a strong need for a different way of speaking of migraine in order to reduce stigma and discrimination [96]. Migraine as every other disease has a large range of severities. The ones who are visible by the general public, by the policymakers or by the payers are often only the less severe ones that can nearly perform in a normal way. The most severe ones are hidden at home with a life completely conditioned by their pain and other symptoms and without any legal or social support [97]. One of the most important current goals is a society that recognizes the disease and the burden of its stigma at home, at the workplace but also in the healthcare systems. Access to innovative treatments should have to be also easier and with less complex protocols since while the affected ones are trying possible not specific treatments, days and days of their life are passing without letting them enjoy a normal life.

## Secondary headaches

Secondary headache disorders are conditions in which headache is a symptom, that usually begins or worsens in parallel with the secondary cause, and improves or ceases when the secondary disorder does [1]. Not all secondary headache disorders are equally threatening, while some causes are relatively benign, others may threaten the patient’s life [98]. In the ICHD-3118 different causes of high-risk headache are listed [1]. If tension-type headache and migraine are the second and third most prevalent disorders worldwide [6**]**, headache as a symptom of a secondary cause may be even more prevalent. The Table 1 depicts the incidence and prevalence of the main disorders listed under the secondary headache categories of the ICHD-3, and the estimated prevalence of headache, along with the 95% confidence intervals (CI).

**Table 1:** Incidence and prevalence of the main disorders listed under the secondary headache categories. Adapted from the Global Burden of Disease study 2017 [6]

Secondary headache category	Prevalence (GBD 2017) (in thousands, 95% CI)	Incidence (GBD 2017) (thousands, 95% CI)	Prevalence of headache (%, 95% CI)
**Trauma injury to the head and/or neck**	46,873 (44984–48,892)	21,652 (19206–24,416)	66 (56–75) (acute phase) [99] 60 (58–63) (at 2 weeks postinjury), 27 (25–30) (at 3 months, 17 (16–19) (at 6 months, 12 (10–13) (at 12 months) [100]
**Cranial or cervical vascular disorder**	104,179 (98454–110,125)	11,931 (11118–12,826)	14 (7–23) (acute ischemic stroke)
**Non-vascular intracranial disorders**	Brain and nervous system cancer: 1706 (1471–1895)	Brain and nervous system cancer: 405 (351–443)	71 (63–77) [101] 48 (38–57) [102]
**Infection**	Meningitis: 10573 (8837–12,552) Respiratory infections: 2187290 (1979143–2,449,761) Malaria: 136085 (126472–145,009) Dengue: 6267 (3416–10,612)	5045 (4435–5878) Respiratory infections: 17942622 (16102037–20,038,445) Malaria: 208768 (170214–257,506) Dengue: 104772 (63759–158,870)	Bacterial meningitis 87 (84–89) [103] Influenza: 91 (90–92) [104] Malaria 74 (68–80) [105] Dengue 76 (69–81) [106]
**Disorder of homeostasis**	Ischaemic heart disease: 126451 (118587–134,706)	Ischaemic heart disease: 10636 (9573–11,794)	14 (11–18) [107]

The true prevalence of secondary headache disorders remains unknown. It varies depending on the region (some headache disorders may be more prevalent in low-and-middle income countries, such as malaria or dengue), the setting (urban versus rural) and the location where the study was conducted (emergency room, outpatient clinics or at a population level). In addition, epidemiological studies must ensure that patients are correctly diagnosed and classified, which may not always be the case.

In a study conducted in Türkiye, Ivory Coast, Chad, Senegal, Sudan, Ethiopia, Morocco, Egypt, Iran from Tatarstan, Turkish Republic of Northern Cyprus, Azerbaijan, and Mongolia 13,794 patients admitted to a hospital or an outpatient clinic were assessed, among which, 4144 (30%) reported headache as the main symptom. The prevalence of secondary headache disorders was 1249/3722 (33%), with no remarkable differences within the studied regions. The most prevalent headache disorders were medication overuse headache (9%), idiopathic intracranial hypertension (3%), and cervicogenic headache (3%) [108].

Due to the generally acute nature, most secondary headache disorders visit the Emergency Department as first contact with the healthcare system. In a study conducted in Colombia, in an Emergency Department during five consecutive weeks, all admissions were screened and in 244/10450 (2.3%), with a proportion of secondary headache disorders of 32% [109]. In another study conducted in an emergency department setting, the proportion of patients with secondary headache disorders corresponded to 11.2% of all headache patients, with 5% of them attributed to disorders with high morbidity and/or mortality [98]. This is particularly relevant, since the education of healthcare providers must be a priority. One study evaluated whether the diagnosis of TTH was accurate in the emergency department, and in 30% of cases, a secondary headache disorder had been misdiagnosed as TTH, reflecting that the need of continuous medical education and training in headache medicine [110–112]. Given the vast prevalence of primary and secondary headache disorders, most patients will be evaluated in primary care, or in secondary care, while tertiary centers should be reserved for difficult to treat or complicated cases, however, in some settings, the number of headache specialists may be insufficient, and most patients are treated by general practitioners or nurse practitioners [113]. In LMIC the lack of resources is even more evidence, and also there is a regional unbalance of health care centers and human resources (e.g. in Morocco far south is the poorest one regarding these elements, followed by east and center. Rural settings are the places most often affected) [113–116]. The differences among geographical regions regarding the prevalences of different secondary headaches is shown in Table 2.

**Table 2:**
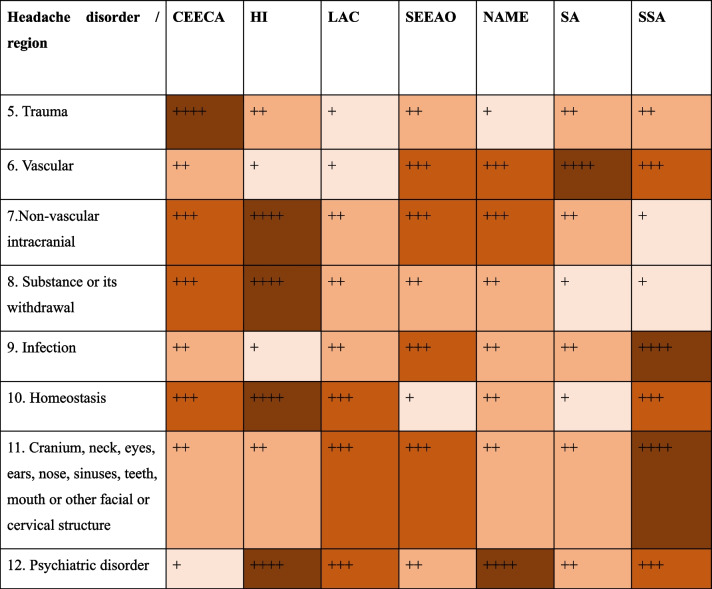
Heat map of estimated prevalence of secondary headache disorders. Darkest colors represent the highest relative prevalence, when compared with the rest of the regions [3–8]

Abbreviations: *CEECA* Central Europe, Eastern Europe and Central Asia, *HI* high-income countries, *LAC* Latin America and Caribbean, *SEEAO* Southeast Asia, East Asia and Oceania, *NAME* North Africa and Middle East, *SA* South Asia, *SSA* sub-Saharan Africa

## Conclusion

Migraine is an extremely common and lifelong disorder. Migraine can affect every domain of life and is associated with a significant disability and poor quality of life, if it is not adequately treated. A correct management should include not only an optimal reduction of pain, in terms of monthly migraine days and intensity of crises, but also the improvement of quality of life, in terms of returning to an acceptable daily routine. These goals can be addressed not only with the relief of pain directly, but also with indirect measures aimed at raising awareness among general clinicians who can manage patients with migraine for the first time, but also among the general population. Social, geographical, and economic differences between the different ethnic groups should be mitigated, even more so after the recent COVID-19 pandemic which led to strong differences in health equity. The demonstration of specific pathophysiological mechanisms, and the use of tailored therapeutic agents, is a great achievement of the modern research of migraine because it could help to reduce the social stigma about this disorder. The recent introduction of new therapeutic targets addressing the CGRP is a significant breakthrough in the management of migraine, and there are interesting results in terms of pain improvement and disability reduction. The high costs of these novel therapeutic agents limit, actually, their routine use only in high-income countries. However, when analyzing the current global health situation in the context of headache medicine, we must also be aware that most likely that headache inequities are likely to persist and worsen due to various factors, including inflation reinforcing health inequities, inaccessible advancing technologies, and the lack of prioritization of academic headache research in resource-rich settings. The ongoing COVID-19 pandemic exacerbates these inequities. Future studies should evaluate the global feasibility of supporting their use in real-world settings and their long-term tolerability. Periodic awareness campaigns should be encouraged to reduce social factors that could stigmatize and underestimate the migraine as a psychosocial disorder and improve headache education at medical schools.

## Data Availability

No datasets were generated or analysed during the current study.
